# Soil microbial communities in contrasting environments show a common core of species linked to *Maytenus senegalensis* shrubs

**DOI:** 10.3389/fmicb.2025.1699694

**Published:** 2026-01-27

**Authors:** Elena Díaz-Santiago, Thiaba D. Sadio, Joseph S. Diéme, Miguel Hurtado-Martínez, Christian Kindler, Esteban Manrique, Francisco I. Pugnaire

**Affiliations:** 1 Departamento de Ecología Funcional y Evolutiva, Estación Experimental de Zonas Áridas, Consejo Superior de Investigaciones Científicas, Almería, Spain; 2 Département d’Agroforesterie, Université Assane Seck, Ziguinchor, Senegal; 3 Real Jardín Botánico, Consejo Superior de Investigaciones Científicas, Madrid, Spain

**Keywords:** co-evolution, core microbiota, plant–soil feedbacks, soil microbial communities, metagenomics

## Abstract

**Introduction:**

The existence of a core microbiota specific to a plant species, or the set of microorganisms shared by all plant individuals of the species, is of utmost importance because of its many conceptual and practical consequences. The core microbiota is assumed to gather the most ecologically and functionally relevant microorganisms associated to a plant in a given environment, presumably establishing positive feedbacks that support its persistence and performance in a plant community.

**Methods:**

We tested the existence of a potential core microbiota in *Maytenus senegalensis* shrubs in two contrasted, distant ecosystems; a dry environment (Almeria, Spain) and a relatively wetter ecosystem (Dakar, Senegal).

**Results:**

Soil microbial community structure widely differed between sites influenced by soil and climate. However, a subset of microbial phylotypes appeared consistently associated to all *M. senegalensis* plants across our two disparate ecosystems while they were absent in the surrounding soil, suggesting the presence of a core microbiota in *M. senegalensis*.

**Discussion:**

Microbiota had an effect on germination that differed between sites, perhaps due to climatic constrains. We show that the assembly of understory microbial communities depends on the plant’s sorting effect on the surrounding soil microbiota, plus some other taxa likely transferred by seeds; this assembly mechanism is relevant for the coevolution of plants and microorganisms, and critical for potential community responses to environmental changes.

## 1 Introduction

Plant–soil interactions are essential components of plant community responses to environmental variability (Chu et al., 2024; Suding et al., 2013) but their role in –and how they respond to—environmental changes remains unclear. These interactions are subsumed in the concept of plant–soil feedbacks. Microorganisms influence many aspects of plant performance (Bever, 2003; Bhattacharyya et al., 2021; Pugnaire et al., 2019; van der Putten et al., 2013) and ecosystem dynamics (Bardgett and Wardle, 2010; de Vries et al., 2013; Pugnaire et al., 2019), playing a main role in nutrient cycling through litter decomposition (Philippot et al., 2024; Rajguru et al., 2024; Sun and Shahrajabian, 2023; Wagg et al., 2014), seed germination (Magaña Ugarte et al., 2024; Pugnaire et al., 2025), and nutrient availability (Mahmud et al., 2020; Pugnaire et al., 2023). Plants, in turn, influence soil microbial communities in many ways, altering the soil environment through, e.g., root exudates and litter (Bever et al., 1997; van der Putten et al., 2013). By mediating how plant diversity affects ecosystem functions, and how soil responses influence plant performance, plant–soil feedbacks provide a mechanistic bridge between biodiversity and ecosystem functioning (Bardgett and Wardle, 2010; Ehrenfeld et al., 2005).

Microbes produce hormones and disease-antagonist agents (Binyamin et al., 2019), remove toxic compounds (Ayangbenro et al., 2022), and promote plant growth (Wardle et al., 2004), influencing processes such as secondary succession (Lozano et al., 2014) and local species diversity (e.g., Janzen-Connell effects), overall modulating ecosystem functioning (Bardgett and van der Putten, 2014). However, and despite increasing evidence, many aspects of the role of microorganisms in these interactions are still little known (Pugnaire et al., 2019; Rodríguez-Echeverría et al., 2013), and a better understanding of these processes is needed to fully understand plant–soil feedbacks, the effects of climate change, and its consequences on these interactions (Pendergast IV et al., 2013).

The rhizosphere and soil in the plant understory embody a distinctive interface between plants and the environment. The microbial community in the soil is regulated either by roots, through the release of thousands of secondary compounds (up to 20% of carbon fixed by photosynthesis; Walker et al., 2003), as well as by leaf litter and other canopy debris. This huge and variable amount of chemical compounds shape the soil microbial community by promoting or preventing the presence of certain microbial phylotypes in both, the plant rhizosphere and understory soil (Ayangbenro et al., 2022; Brinkman et al., 2010), shaping the composition of their associated microbial communities (Hortal et al., 2013) in a process that could be termed as true co-evolution. In addition, the special set of environmental conditions created by the canopy, in terms of radiation, temperature, humidity, or litter accretion (Cavieres et al., 2014) further contributes to shape microbial community composition (Hortal et al., 2013).

It has been proposed that all plants of the same species may share a set of common microorganisms, referred to as the core microbiota (Vandenkoornhuyse et al., 2015). Such core microbiota is expected to gather the most ecologically and functionally relevant microorganisms associated with a plant species in a given environment (Compant et al., 2019; Durán, 2024; Neu et al., 2021), assuming the establishment of positive plant-microbial feedbacks that support plant persistence and performance in a community (Bever et al., 2012; Pugnaire et al., 2019). However, whether there is such a core microbiota remains unclear.

In distant plant communities it could be expected that soil microbial communities associated with a plant species differ as soil, climate, and bedrock differ (Durán, 2024). Therefore, the presence of common microbial taxa should be highly relevant. Clarifying the presence and origin of the core microbiota could provide insights into a plant’s competitive abilities and overall performance in a community.

Here, we aimed at comparing plant–soil relationships in *Maytenus senegalensis* (Lam.) Excell. (Celastraceae) shrubs growing in a dry ecosystem (Almeria, Spain) and in a relatively wetter ecosystem (Dakar, Senegal), trying to determine similarities and differences in their associated soil microbial communities; whether they share a core microbiota, and whether plant–soil interactions vary depending on environmental factors. This would allow us to understand how soil microbial communities assemble and their relationships with the plant.

We hypothesized that plants in both environments will select certain microbial phylotypes from their surrounding soil to form a specific microbial community, and that *M. senegalensis* shrubs from the two contrasted, distant environments will share a group of microbial phylotypes (the core microbiota), that can be found in soil regardless of differences in soil properties and climate. We also expected that soil microbial communities associated with *M. senegalensis* understories would feedback, enhancing seed germination more than communities from gaps; i.e., there is a home-field advantage provided by local soil communities (Magaña Ugarte et al., 2024; Pugnaire et al., 2025).

## 2 Materials and methods

### 2.1 Characterization of microhabitats and species

The spatial scale over which the host is sampled can have a strong influence on the composition of the core. We thus selected two *Maytenus senegalensis* communities across two continents in contrasted, distant environments with different climate regimes and soil types. Field sites included a coastal community in El Ejido, Almería, SE Spain, at the N edge of the species’ distribution area, and in the Mbao forest and near the village of Noflaye, in the Dakar region of Senegal, close to the southern edge of *M. senegalensis* distribution area. Both communities differ in rainfall and temperature (Table 1). El Ejido, with a Mediterranean semi-arid climate, hosts the best example of this specie in Spain. It has a pronounced dry season from June to September and scarce rain most years (mean 200 mm), with January (mean temperature 12.5 °C) and August (mean temperature 28.0 °C) as the coldest and warmest months, respectively (BSk in Köppen classification). Soils are calcic regosols and cambisols, with a mixed clayey substrate with a calcic hardpan close to the soil surface. The region of Dakar has a hot semi-arid climate influenced by the proximity of the ocean (Köppen climate classification: BSh), with a short rainy season between July and October. Mean annual precipitation (MAP) is 421.9 ± 24.7 mm, and mean annual temperature (MAT) is 24.17 °C, with small seasonal variation. Soils are sandy and of alluvial origin.

**Table 1:** Location and main data of our two field sites including mean annual precipitation (MAP), mean annual temperature (MAT), and soil chemistry including pH, organic matter (OM), nitrogen (N), phosphorus (P), and potassium (K), C/N and N/P ratios, as well as quantitative PCR results.

	El Ejido (Spain)		Dakar (Senegal)	
	Understory	Gaps	Understory	Gaps
Latitude	36°45’N		14°45’N	
Longitude	2°47’W		17°11’W	
Elevation (m)	83		850	
MAP (mm)	220		421.9	
MAT (°C)	18.3		24.7	
pH	7.70 ± 0.07^b^	8.12 ± 0.09^a^	5.75 ± 1.91^c^	6.10 ± 0.06^c^
OM (g/kg)	6.80 ± 0.57^a^	2.90 ± 0.13^b^	3.36 ± 1.77^b^	1.35 ± 0.10^c^
C (g/kg)	5.90 ± 0.81^a^	5.36 ± 0.81^ab^	3.34 ± 0.81^bc^	2.11 ± 0.86^c^
N (g/kg)	0.39 ± 0.05^a^	0.14 ± 0.05^b^	0.23 ± 0.05^b^	0.10 ± 0.05^b^
P (g/kg)	0.07 ± 0.01^a^	0.04 ± 0.01^b^	0.02 ± 0.01^b^	0.03 ± 0.01^b^
K (g/kg)	0.74 ± 0.03^a^	0.25 ± 0.03^b^	0.05 ± 0.03^c^	0.05 ± 0.03^c^
C/N	20.09 ± 3.70^b^	40.02 ± 3.51^a^	14.43 ± 3.51^b^	20.09 ± 3.70^b^
N/P	6.62 ± 1.51^b^	3.98 ± 1.51^b^	12.22 ± 1.51^a^	7.23 ± 1.59^b^
Bacteria (copies/g of soil)	2.0E+10 ± 1.2E+09^a^	8.2E+09 ± 1.2E+09^b^	4.8E+09 ± 1.2E+09^ab^	2.8E+09 ± 1.3E+09^c^
Fungi (copies/g of soil)	7.4E+07 ± 6.9E+06^a^	4.4E+07 ± 6.9E+06^b^	1.6E+07 ± 6.9E+06^c^	1.1E+07 ± 7.2E+06^c^

Values for MAP and MAT are annual means for the period 1991–2021.

*Maytenus senegalensis* is a thorny shrub up to 3 m tall with coriaceous perennial leaves, an intricate spherical canopy, and deep roots distributed in the southeast of the Iberian Peninsula and the Maghreb and tropical areas of Africa, the Middle East, the Arabian Peninsula and the Irano-Turanian region. In Senegal it is a common specie with important economic and sociocultural roles (da Silva et al., 2011; Sadio, 2024), while in Europe it is limited to a few coastal systems in SE Spain, protected under the Natura 2000 act.

### 2.2 Seed sampling

*Maytenus senegalensis* seeds were collected from both locations in Spain and Senegal. As fruits are small and dehiscent, we harvested them before valves opened. Once at the lab, fruits were covered with mosquito nets and dried to facilitate valve opening. Seeds were then extracted and used in the germination experiment.

### 2.3 Soil sampling

Soil samples (~1 kg) were collected in the understory of *M. senegalensis* shrubs, including bulk and rhizosphere soil, and in paired gaps nearby. In each field site we collected soil samples from the top 10 cm layer in three points under the canopy of 10 randomly selected *M. senegalensis* shrubs and in 10 nearby gaps. A subsample was collected in an Eppendorf tube for DNA extraction and another one collected with 20 mL Falcon tubes for physicochemical analyses. Soils were kept in a cool box in the field and brought back to the lab, were samples for DNA extraction were stored at -5 °C and then to -80 °C until they were processed. Other samples were kept refrigerated until the setup of the experiment to avoid microbial degradation. Soil samples included bulk soil but also rhizosphere soil, as roots were often superficial. All analyses were carried out on individual samples.

Soil nutrients were determined at the CEBAS-CSIC ionomics lab (Murcia, Spain), including total C and N content using a C/N analyzer (LECO Truspec, St. Joseph, MI, USA). Other elements were determined after acid digestion with an inductively coupled plasma (ICP) emission spectrometer (ICAP 6500 DUO; Thermo Scientific, Wilmington, DE, USA). pH was measured with a pH-meter (Hach Sension+ PH3, USA) in a 1:2.5 (w:v) water solution, and organic matter by dry combustion at 550 °C for 4 h.

### 2.4 Seed germination

We used 0.5 L pots filled with either understory or gap soil from each of the 10 shrub selected in Spain and 15 in Senegal. To assess the effect of soil microbiota in the germination process, pots were autoclaved at 180 °C for 120 min and seeds were sterilized by keeping them for 5 min in a 4% sodium hypochlorite solution, then rinsed for 2–3 min with distilled water, and submerged in 75% ethanol for 2 min; then, rinsed again with distilled water and plotted dry with paper towels. Soils were not sterilized to allow natural soil microbiota differ between treatments (understory and gaps) while other environmental factors were equal for both treatments. Therefore, the outcome would only be attributable to soil differences. Pots in Spain were seeded on December 2022, watered weekly with 100 mL of deionized water per pot, and root emergence was recorded every week until germination stopped in June 2023. In Senegal, the experiment took place between September and December 2022 under similar experimental conditions. Pots were kept in rainout shelters under natural conditions of radiation and temperature, and were rearranged every other week to avoid environmental gradients. To test for differences between treatments, we used a linear mixed model (LMM) with country and microhabitat (understory, gap) as fixed factors and replicate as random factor. Normality of residuals and homogeneity of variances were assessed by graphical inspection of residuals; when these assumptions were not met, we used a model correction for heterogeneity of variance (varIdent). *Post hoc* differences were tested with Fisher least significant difference tests. Statistical analyses were performed with R (version 4.1.2) using the InfoStat statistical package (Di Rienzo et al., 2020).

### 2.5 DNA extraction and quantitative PCR analysis of soil microbial communities

DNA was extracted from 250 mg of soil using the DNeasy PowerSoil Pro® Kit (Qiagen, Venlo, Netherlands) following the manufacturer’s protocol. DNA concentrations were quantified using a Qubit Fluorometer (Thermo Scientific, USA) and NanoDrop. The samples were stored at −80 °C.

Quantitative PCR (qPCR) was performed to quantify the abundance of microbial marker genes for bacteria and fungi in the soil DNA extracts. The primers used for the qPCR analyses were EUB338f (5′-ACTCCTACGGGAGGCAGCAG-3′) and EUB518r (5′-ATTACCGCGGCTGCTGG-3′) for bacteria (Fierer et al., 2005), and ITS1f (5′-TCCGTAGGTGAACCTGCGG-3′) ((Fierer et al., 2005; Gardes and Bruns, 1993) and Earth Microbiome Project) and ITS2 (5′-GCTGCGTTCTTCATCGATGC-3′) (Earth Microbiome Project) for fungi.

qPCR amplifications were performed using a SYBR® Green-based method (Sigma-Aldrich, USA) on a CFX96™ Real-Time PCR Detection System (Bio-Rad Laboratories, USA). Standard curves were generated in each assay using 10-fold serial dilutions of target DNA stock solutions. The 20 μL reaction mixture consisted of 10 μL of 2X PowerUp™ SYBR™ Green Master Mix (Applied Biosystems, USA), 0.4 μL of each primer (20 μM), 10–100 ng of template DNA, and nuclease-free water (Ambion Thermo Fisher, USA).

Amplification conditions were as follows: an initial denaturation at 95 °C for 2 min; for bacteria, 35 cycles of 95 °C for 15 s, 55 °C for 15 s, and 72 °C for 1 min; for fungi, 40 cycles of 95 °C for 2 min, 60 °C for 15 s, and 72 °C for 1 min, followed by a melt curve analysis from 60 °C to 95 °C with a 0.5 °C increment. All reactions were performed in triplicate, including DNA extracts, standard curves, and negative controls.

PCR efficiency, calculated from the slope of standard curves obtained from 10 fold serial dilutions of a DNA template, ranged from 86 to 95% for prokaryote assays and from 81 to 87% for fungal assays. All standard curves showed high linearity with R^2^ values exceeding 0.99. The specificity of the amplified products was confirmed by melting curve analysis.

### 2.6 DNA sequencing

DNA samples were sent to the Integrated Microbiome Resource (IMR) at Dalhousie University, Canada, for library preparation and Illumina MiSeq sequencing. The V4-V5 region of the ribosomal RNA gene (16S) was sequenced to characterize prokaryotic communities using the primer pair 515FB-926R (Walters et al., 2015), while the internal transcribed spacer (ITS2 region) was sequenced to characterize fungal communities using the primer pair ITS86F-ITS4R (Op De Beeck et al., 2014). Detailed sequencing protocols can be found on the IMR website.^1^ To ensure data quality, a commercial mock community sample (ZymoBIOMICS Microbial Community Standard, Zymo Research, Orange, California), was included in the sequencing pool as a positive control and a DNA extraction kit as a negative control sample. Raw sequences are available at PRJNA1223696.^2^

### 2.7 Bioinformatics’ pipeline

Sequence processing was performed with *QIIME2* (v2023.5, Bolyen et al., 2019), whereas, further analyses were performed in R (v.4.4.1). A comprehensive report of all results was generated using the *rmarkdown* CRAN-package (v.2.28), which allows the seamless integration of code, results, and explanatory text into a single, reproducible HTML document that can be easily viewed in a web browser (code and *rmarkdown* templates are available at GitHub https://github.com/Elenadisa/M.senegalensis-core-microbiota). Other packages were used for specific analyses. For data management and visualization, we used *multcompView* (v.0.1–10), *dplyr* (v.1.1.4), *ggplot2* (v.3.5.1), *ggpubr* (v.0.6.0), *magrittr* (v.2.0.3) and *ggVennDiagram* (v.1.5.2). The final HTML reports were customized using the *knitr* (v.1.48), *kableExtra* (v.1.4.0), and *pander* (v.0.6.5) CRAN-packages. To import *QIIME2* qza files into R we used the package *qiime2R* (v.0.99.6).

### 2.8 Metabarcoding

Pair ended sequences were processed using the *QIIME2* pipeline for 16S and ITS sequences. The *q2-cutadapt* plugin (Martin, 2011) was used to trim primers and correct for primer extension in ITS sequences, due to the variable length of these amplicons (Morillo et al., 2022). The *DADA2* plugin (Callahan et al., 2016) was employed for trimming and denoising, which involved quality control of the sequences, truncation of the reads, merging of R1 and R2 reads, generation of amplicon sequence variants (ASVs), and removal of potentially chimeric sequences.

To ensure accurate sequencing, both a negative control (kit control) and a positive control (a known composition of 8 bacterial and 2 fungal phylotypes) were included alongside the samples. The negative control showed minimal amplification. The positive control successfully identified 7 of the 8 bacterial genera and both fungal genera (results in HTML reports available at GitHub).

Taxonomy was assigned using the Naïve Bayes machine-learning classifier implemented in *QIIME2’s* q2-feature-classifier (Bokulich et al., 2018). For 16S and ITS data, the pre-trained SILVA (v.138–99) (Quast et al., 2013) and UNITE (v9 dynamic) (Kõljalg et al., 2005) databases were used, respectively. ASVs assigned to chloroplasts and mitochondria (for 16S data), as well as non-fungal eukaryotic lineages (for ITS data), were removed, along with ASVs that could not be classified at the phylum level.

### 2.9 Diversity of the edaphic microbiota

Compositional analysis was performed with *phyloseq* R-Bioconductor package (v.1.48.0, McMurdie and Holmes, 2013). We have performed a data normalization with Cumulative Sum Scaling (CSS) algorithm before to perform further analysis with the R-Bioconductor package *microbiomeMarker* (v1.10.0, Cao et al., 2022). We chose not to apply rarefaction methods, as they can lead to an incomplete or inaccurate representation of the microbial communities by removing tens or hundreds of thousands of sequences from individual samples (Neu et al., 2021), potentially distorting diversity and abundance patterns.

Moreover, we performed a differential abundance analysis with the R-Bioconductor *DESeq2* package (v.1.44.0, Love et al., 2014) to see if there are differences between understory and gap samples. We considered that differences in abundance are significant with an adjusted *p*-value < 0.05. In genera with significant differences in abundance between gap and understory, a functional analysis was carried out. The R-Bioconductor package *microeco* (v.1.15.0) was used along with the FAPROTAX database (Louca et al., 2016) for prokaryotic data and the FUNGuild (Nguyen et al., 2016) for fungal data.

Diversity indices were calculated to assess the differences in the microbiota community structure across each experimental condition. To this end, we filtered every ASV that are in less than the 10% of the samples. Chao α-diversity index was assessed with a Kruskal Wallys test and with a BH *p*-value correction. For a β-diversity analysis, Bray–Curtis dissimilarity index was calculated to evaluate the linkage between the microbiota and experimental conditions. Moreover, to assess the significance we have performed a Permutational Analysis of Variance (PERMANOVA) test with *vegan* (v2.6–6.1) and *pairwiseAdonis* R packages (v.0.4.1).

### 2.10 Identifying the core microbiota

There is no consensus in the scientific literature on the optimal strategy for core microbiota identification (Neu et al., 2021). Given this lack of standard methodology, we aimed to provide a clear and reproducible framework that could serve as a reference for future studies. To investigate the soil core microbiota associated to *M. senegalensis* we implemented an abundance–occurrence approach (Neu et al., 2021) to differentiate a stochastic from a deterministically selected core, ensuring that the scale of spatial sampling was adequate to reliably capture macroecological relationships (Neu et al., 2021). In addition, we compared our understory soil data with local soil data not influenced by our species to further assess the uniqueness of the core microbiota under *Maytenus senegalensis* shrubs. To this end, ASVs were agglomerate at the genus level. To minimize the inclusion of potential sequencing artifacts an abundance filter was applied. There is no consensus to stablish a threshold for the abundance. Therefore, to explore abundance patterns of microbial genera in gaps and understories, a scatter plot was produced to check for the abundance of genera in soil samples; we applied different thresholds of relative abundance (≥ 0.2, 0.5, 1, 5%) at the genus level to focus on taxa with significant presence and better compare microbial communities across microhabitats. Then, genera found in gap samples were discarded from understory data. This step will ensure that only genera linked to the shrub were selected, identifying obligate relationships between host and core microbes. Finally, to determine core members, the proportion of samples over which a microbial genus must occur to be considered core was conservatively set at 100% of understory samples in both sites, Senegal and Spain, and as null model we used the 100% absent in gap soils.

## 3 Results

### 3.1 Differences between sites and microhabitats

The two plant communities featured contrasting climatic conditions. El Ejido in Spain is cooler and drier (mean annual temperature, 18.3 °C; mean annual precipitation, 220 mm) than Dakar in Senegal (24.7 °C, 421.9 mm, respectively) (Table 1). Soils also differed in several properties. For instance, pH in Senegal was slightly acidic while in Spain it was slightly basic (Table 1). Differences were more important concerning OM, which in gaps in Spain were twice as much as in Senegal, most likely because soils in the latter were quite sandy. Organic matter in the understory more than doubled OM in gaps in both Spain and Senegal (Table 1). Most other nutrients followed a similar pattern, except P and K in Senegal, which were similar in gaps and understories (Table 1). As a consequence, there were big differences in nutrient ratios (C/N and N/P), which along with climate differences, likely contributed to differences in microbial and plant population dynamics.

### 3.2 Differences between sites and microhabitats

In both field sites, qPCR data show that microbial populations were much higher in the understory than in gaps (Table 1); however, this difference was only significant for fungi in Spain samples. We found higher numbers of prokaryote and fungi of different genera in Senegal than in Spain, although differences were not significant between gap and understory microhabitats within the same field site (Supplementary Table S1).

**Figure 1:**
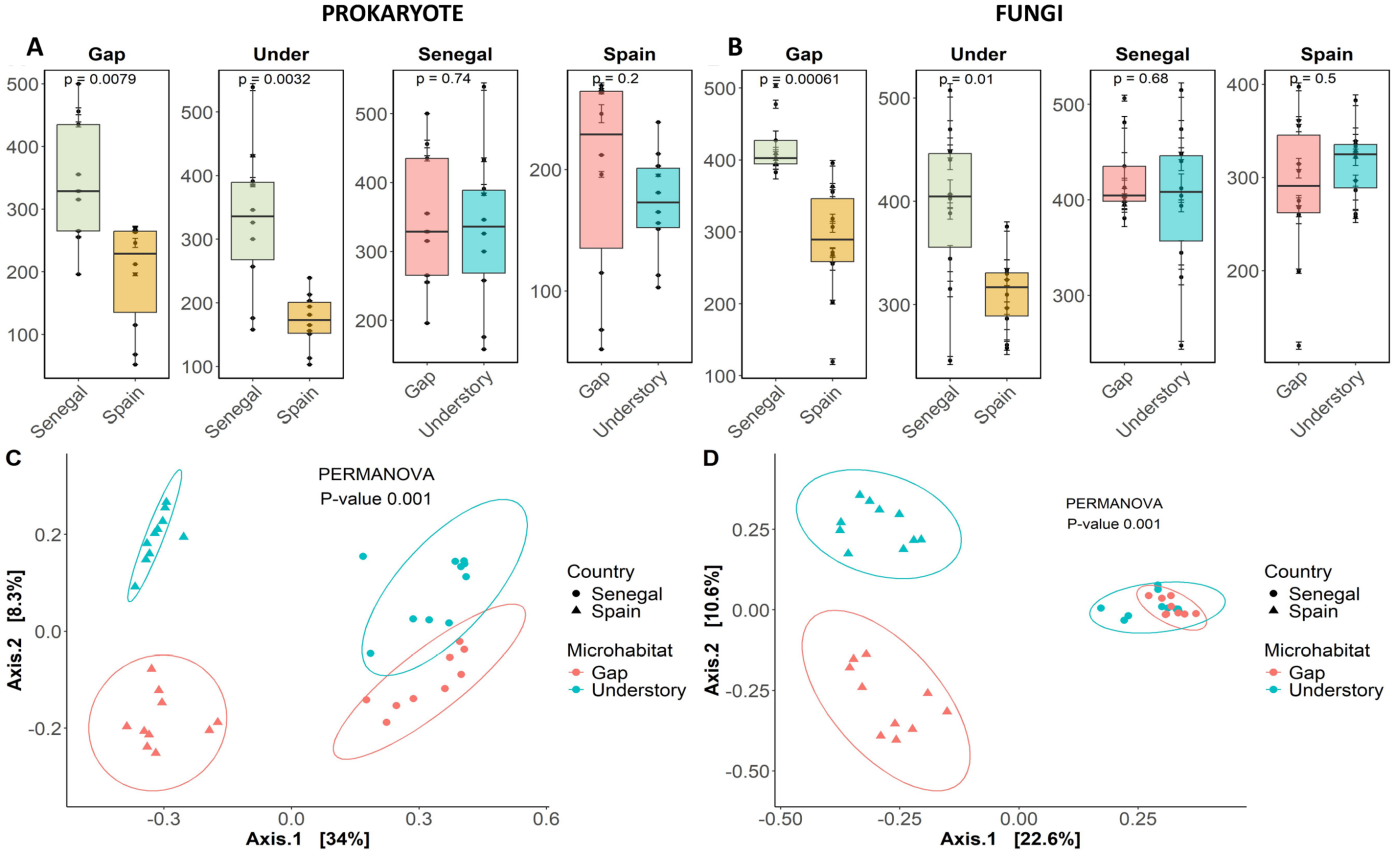
Boxplot showing α-diversity data (Chao index) of prokaryote **(A)** and fungi **(B)** in *Maytenus senegalensis* gap and understory soils from Senegal and Spain. Principal coordinate analysis of β-diversity based on Bray–Curtis dissimilarity of prokaryote **(C)** and fungi communities **(D)** in Spain (triangles) and Senegal (dots) understories (blue) and gaps (orange). PERMANOVA shows significance of differences between groups.

Chao’s α-diversity index showed significant differences in gap and understory diversity between Spain and Senegal for both, prokaryote and fungi (Figures 1A,B), being diversity overall higher in Senegal. However, diversity was similar between understories and gaps in both sites and for both microbial groups (Figure 1), suggesting that climate has a stronger effect on microbial richness than the presence of the plant itself.

**Figure 2:**
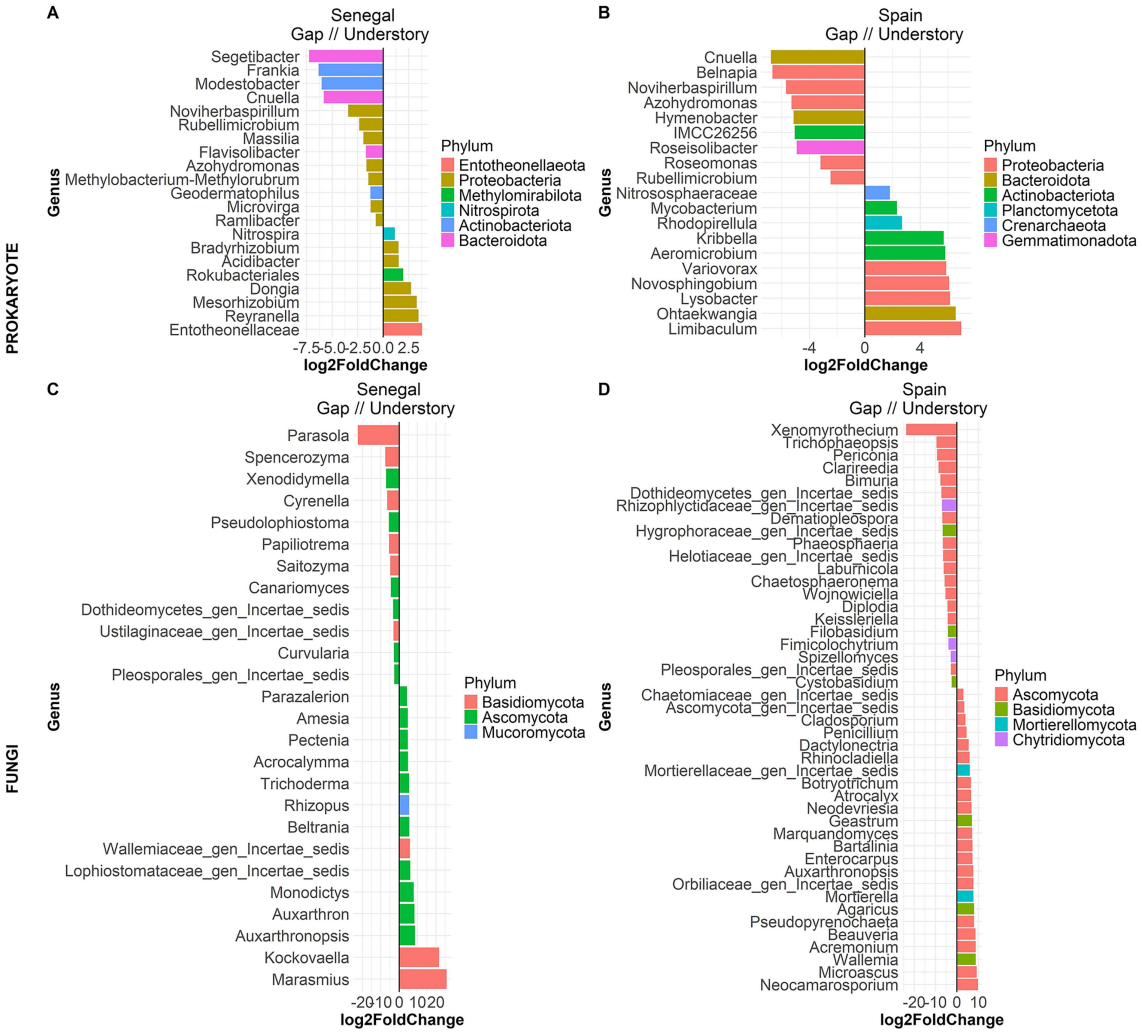
Differential abundance analysis comparing Prokaryote **(A-B)** and Fungi **(C-D)** genera in different microhabitats with DESeq2. Positive log2FoldChange values show that the genus is more abundant in the understory than in gaps, and viceversa. Only genera that show significant differences between the groups (adjusted *p*-value < 0.05) are shown in the bar chart. Colors show phylum level.

PERMANOVA results for prokaryote (Supplementary Table S2) and fungi (Supplementary Table S3) showed major differences in microbial composition between sites based on Bray Curtis dissimilarities, showing contrasting microbial communities between sites. PCoA grouped sites and microhabitats in different clusters (Figures 1C,D), being those from Senegal closer to each other than in those from Spain. This fact is more evident in Senegal fungal communities from understory and gaps, which overlapped (Figure 1D), suggesting that in wetter environments, plants might have a less pronounced effect on soil microbial communities than in arid ones (Figure 2).

**Figure 3:**
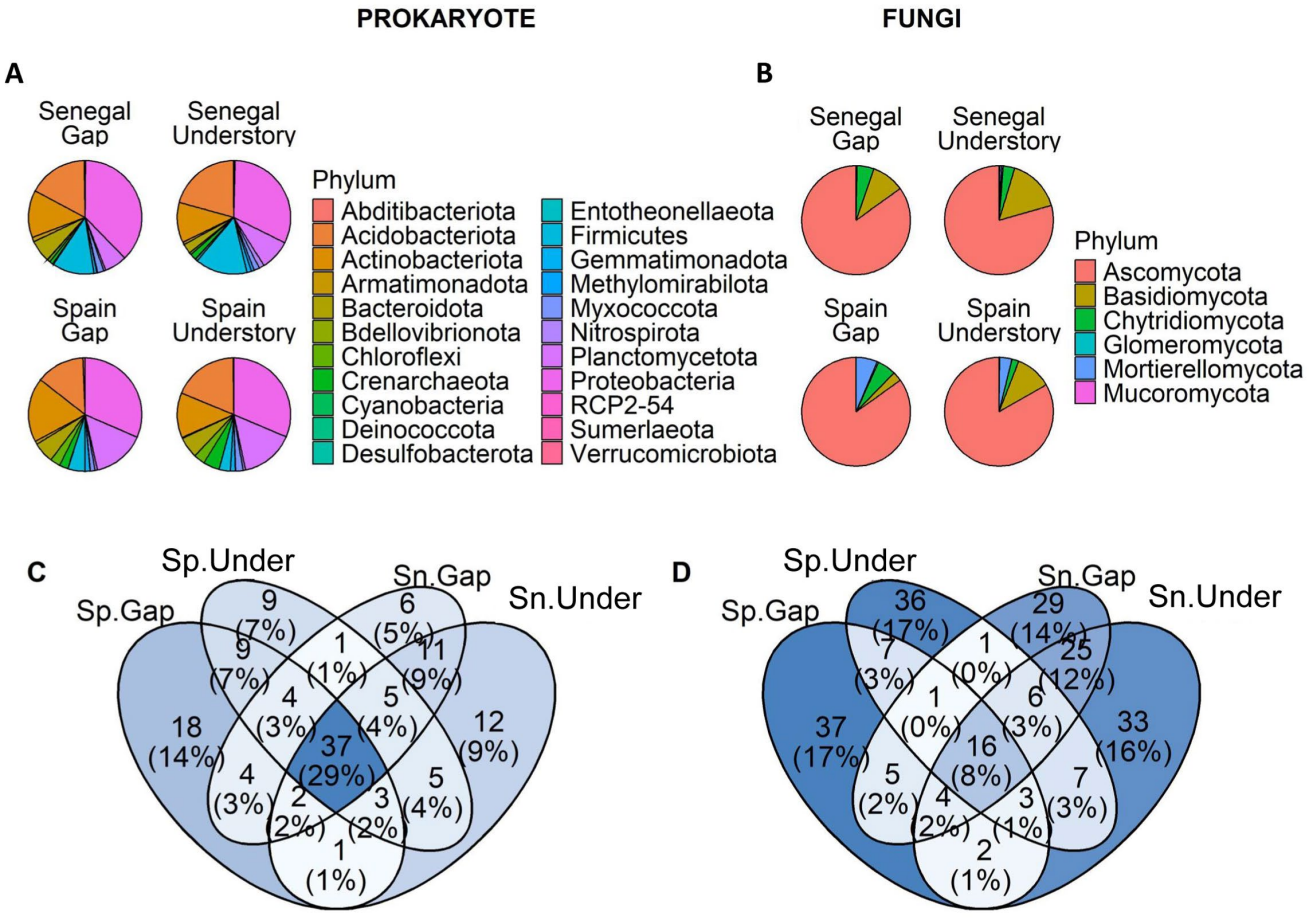
Phylum relative abundance. **(A)** for prokaryote. **(B)** for fungi. Venn diagram including the number of prokaryote **(C)** and fungi **(D)** genera in soil samples from different countries and microhabitats (understory, gaps), and their relative proportion (%). *Maytenus senegalensis* understories in Senegal and Spain shared a number of prokaryote and fungi genera, while at the same time hosted site-specific phylotypes. Sp = Spain, Sn = Senegal, Unde = Understory.

Despite differences in α and β diversity, there were phylotypes shared between sites and between microhabitats both at the phylum (Figures 3A,B) and genus (Supplementary Figure S1) level. Although understory communities in Spain and Senegal showed a number of site-specific genera, there were prokaryote and fungi genera common to both sites only with slight differences in abundance (Supplementary Figure S1). Moreover, the genera shared between understory and gap samples significantly differed in abundance (Figure 3; Supplementary Figure S2). By applying an abundance filter to each microhabitat, we found that, among all genera exclusive of *M. senegalensis* understories. As we expected, increasing the threshold reduced the total number of genera retained and changed the identity of the exclusive genera between conditions (Supplementary Table S4). At a threshold of 0.2% there were 5 prokaryotes and 7 fungal genera shared between Senegal and Spain (Figures 3C,D). Of these genera, 1 prokaryote and 2 fungi genera were present in all samples. The prokaryote genus in all samples was *Reyranella*, and *Auxarthron* and *Agaricus* among fungi.

**Figure 4:**
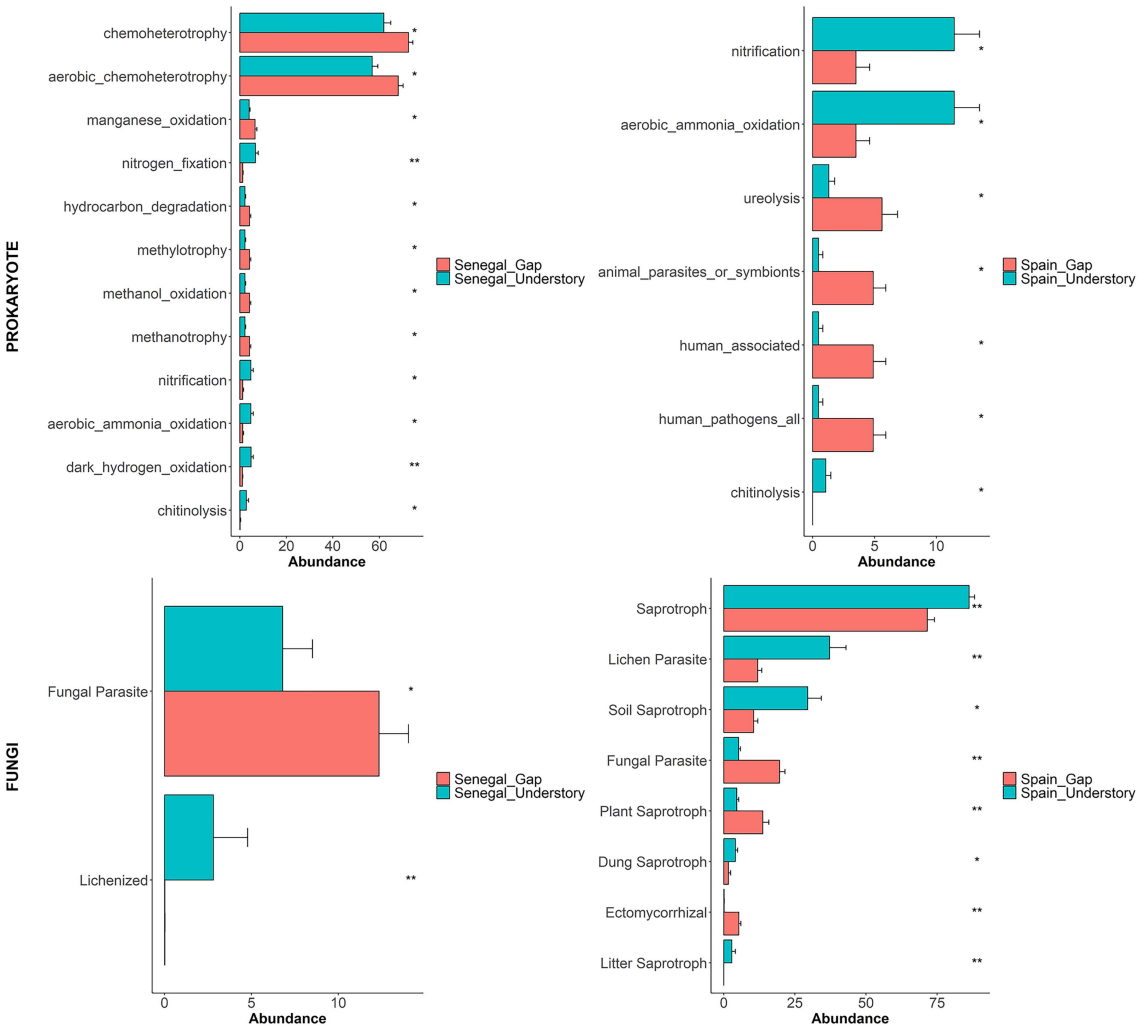
Microbial functional trait analysis in shrub understory and gap microhabitats across Spain and Senegal. Functions were inferred from FAPROTAX for prokaryotes and from FUNGuild for fungi. Genera that were differentially abundant according to DESeq2 analyses (adjusted *p* < 0.05) were used.

Testing the potential functions that enriched taxa could have in the *DESeq2* analysis, we found that, despite climatic differences, the microorganisms taxa enriched in understory communities have higher activity in the nitrogen cycle (nitrification, fixation, ammonium oxidation), in decomposition processes (chitinolysis, saprotrophy), and symbiotic associations (lichenized / lichen parasites), which indicates that they are more stable and rich microhabitats (Figure 4). Gaps, by contrast, were dominated by microorganisms that metabolize simple compounds or are related to oxidative stress (chemoheterotrophs, methylotrophs, ureolytic bacteria) and by opportunists (fungal parasites and plant pathogens).

### 3.3 Seed germination

**Figure 5:**
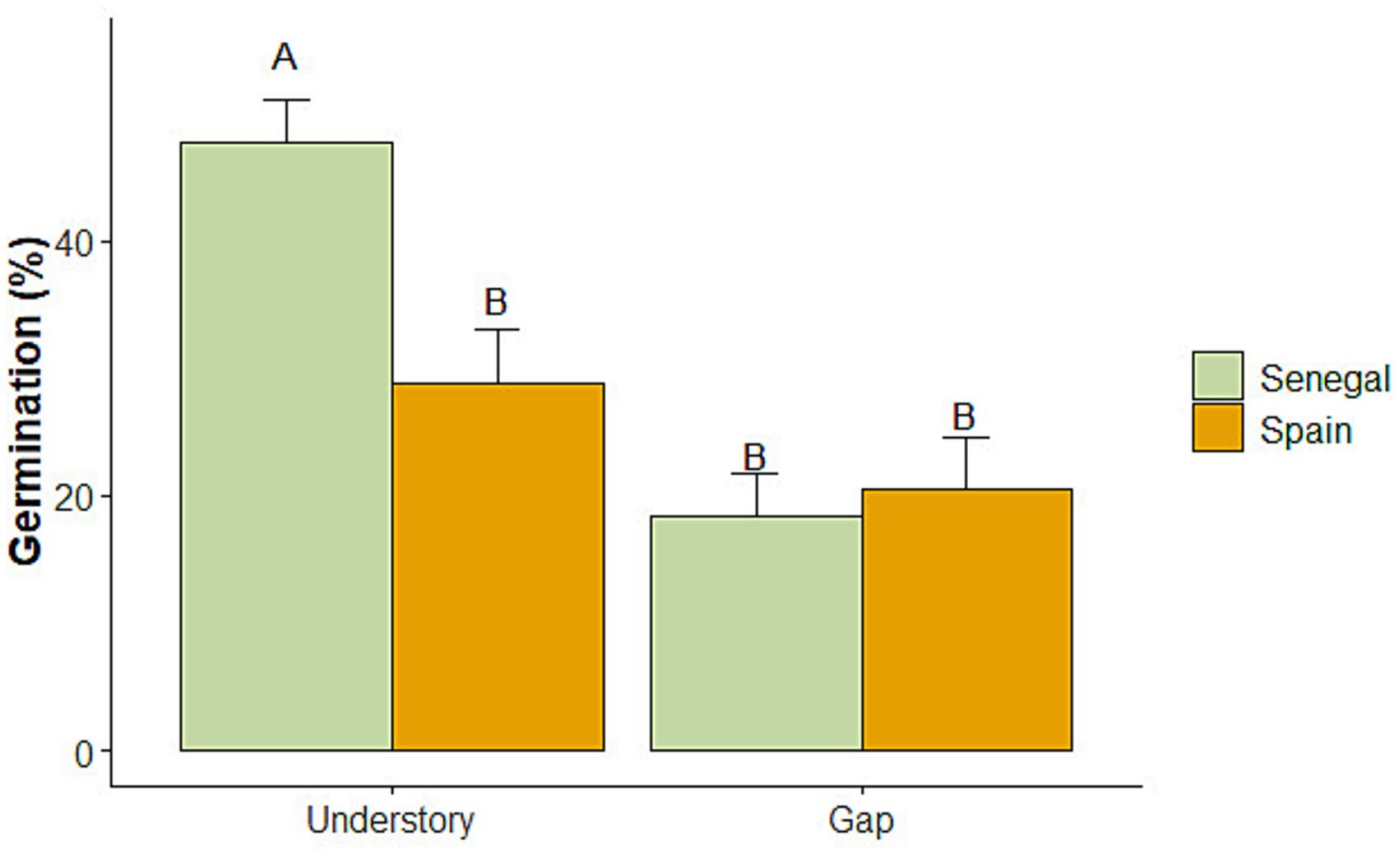
Seed germination on understory and gap soils from Senegal and Spain. Bars are mean values (*n* = 10); lines above bars show the standard error. Significant differences between groups were determined by linear mixed models (LMM) followed by a LSD test. Different letters above bars show significant differences between groups (*p* < 0.05).

Overall seed germination was slightly higher in Senegal than in Spain soils (33.00 ± 2.53% vs. 24.67 ± 3.08%, respectively, *p* = 0.03), and much higher in the understory than in gaps (38.27 ± 2.80% vs. 19.40 ± 2.80%, *p* < 0.0001), being always higher in Senegal (Figure 5). Understory soil characteristics appear to be aligned with key soil processes, including nutrient mineralization and the provision of compounds (such as N fixation, saprotrophy, or ammonia oxidation) that could create more favorable microenvironments for seed germination by increasing nutrient availability and overall soil quality.

## 4 Discussion

As expected, we found that germination was overall higher in understory than in gap soils, suggesting a co-evolutionary process between plants and soil microorganisms leading to positive feedbacks that improve some aspects of plant performance, like seed germination (Magaña Ugarte et al., 2024; Pugnaire et al., 2025). In our experiment, differences in germination between gap and understory soils could only be attributable to soil communities, since climate was common. However, it is important to highlight that seed germination is also influenced by other uncontrolled factors such as seed size, genotypic variation, or soil texture.

### 4.1 Microbial communities structure and functions

Soil microbial communities were more diverse in Senegal than in Spain, and had a higher number of genera, which might contribute to the higher germination rates in Senegal. However, despite the similarity of both microbial communities, germination in the understory of Senegal shrubs was much higher than in gaps. By contrast, microbial soil communities in Spain were quite different between understory and gaps, although germination was rather similar. Such differences could be a consequence of limited co-evolution between plants and microorganisms driven by climate, which may have restricted the richness, diversity and community composition of soil microorganisms in the driest plant community, Spain. Mediterranean semiarid communities, with long, dry summers and mild, moist winters, pose a severe threat to plants and soil organisms, which must adapt to several months of desert-like conditions in summer. Thus, the richer and more diverse communities in Senegal host more phylotypes likely enhancing seed germination (Pugnaire et al., 2025). However, it is important to note that the effects attributed to climatic factors such as temperature and precipitation may be partially masked by soil type and its physicochemical properties, which could exert a strong influence on the formation of soil communities.

These results align with previous reports that addressed soil microbes as key drivers of seed germination showing, for instance, that non-sterilized seeds have higher germination rates than sterilized ones (Magaña Ugarte et al., 2024). Our data support the existence of a ‘home-field advantage’ where plants benefit more from interacting with local microbes than with those from other environments. In our experiment, soil microbial communities from wetter environments enhanced seed germination more than those from drier sites, suggesting that coevolution between plants and soil microbes may be constrained in harsh climates (Pugnaire et al., 2025). These data support the idea that microbial diversity and local adaptation influence plant–soil feedbacks and seed germination patterns.

Differences in microbial phylotypes between gaps and understories, and between Senegal and Spain soil communities, evidence the overall role of soil and climate in shaping these communities. Although the total number of genera did not differ significantly in terms of β-diversity in the same country (Supplementary Table S3), there are significant differences in the phylotype composition between microhabitats (Figure 1), which means that communities in the two sites have a similar number of phylotypes, but their abundance is different. Despite the contrasting climatic and edaphic conditions, understory communities of *M. senegalensis* in both countries have similar functional profiles. Both communities are enriched in microorganisms related to the nitrogen cycling as well as with chitin degradation, which indicates active nutrient cycling. Similarly, fungi in the understory were dominated by saprotrophic and lichenized guilds, which means that the community is enriched in organic matter decomposition and stable symbiotic interactions. This results show the strong influence microhabitats have on soil microbial functions. Interestingly, α- and β-diversity are significantly different between understory and gap communities in Spanish soils, as there are differences between Senegal and Spain understories, which seems to suggest that aridity may cause marked differences between soil microbial communities (Supplementary Figure S1). Our data show that climate drives soil microbial community structure, potentially impacting ecosystem functioning (Pugnaire et al., 2019; Vanisree et al., 2022). Given that soil microbes play key roles in nutrient cycling and plant–soil interactions, shifts in microbial composition due to climate differences—and potentially climate change—could have effects on ecosystem stability and resilience.

### 4.2 Core microbiota

However, despite significant differences in microbial community composition between these contrasting environments, we could identify a subset of microbial taxa that were consistently present in *M. senegalensis* understory soil in both, Senegal and Spain. This presence suggests that, beyond the influence of environmental variables, certain microbial taxa form a core microbiota in the plant that is reflected in the soil across geographically distant populations. Thus, some prokaryotic and fungal genera were present in all understory *M. senegalensis* soil samples from both countries, but absent in gaps. In other cases, some phylotypes were much more abundant in the understory than in gaps. These common microbial phylotypes suggest the existence of a core microbiota in the plant, shared by all *M. senegalensis* plants analysed, even though they are separated by thousands of kilometres. As such, this core microbiota appears to be independent from soil and climate. Deciphering the core microbiota is rather important, as these microorganisms might be involved in major plant functions such as nutrition or the resistance to biotic and abiotic stresses (Neu et al., 2021; Pershina et al., 2018; Vandenkoornhuyse et al., 2015), and may be critical for plant performance (Lv et al., 2023). It has been proposed that the core comprises functional gene clusters rather than individual taxa (Neu et al., 2021), reflecting critical functional relationships with the host (Shade et al., 2017).

The presence of specific microbial taxa in *M. senegalensis* understories suggests additional ecological roles that may influence plant fitness and development. For instance, *Reyranella*, a bacteria genus in Phylum *Pseudomonadota*, is involved in nutrient cycling (Jia et al., 2022). The fungi *Auxarthron*, found in the core, includes keratinophilic species and its role in soil is not fully understood, but have been reported to produce secondary metabolites with antimicrobial properties (El-Zehery et al., 2024; Kim et al., 2019). The genus *Agaricus* includes species involved in lignocellulose biodegradation processes (Sánchez, 2009), and some phylotypes are able to stablish symbiotic relationships with bacteria (Pawlowska, 2024). Overall, microorganisms exclusively found in *M. senegalensis* understory soils are linked to processes that improve access to nutrients by enhancing the nutrient cycling, suggesting a coevolution between plants and microbial communities in nutrient-limited environments. However, the question of how these microbial taxa are selected and maintained by the plant requires further exploration.

Our understanding of the core microbiota and its composition and function in natural environments remains limited, as environmental factors introduce significant complexity. While a standardized approach can enhance consistency across studies, achieving a comprehensive understanding of the core microbiota is still a challenge. However, gaining insights into the core microbiota holds considerable ecological and practical potential to understand plant–soil feedbacks.

In conclusion, the assembly of understory microbial communities depends on the plant’s sorting effect on the surrounding soil microbiota, in addition to other taxa likely transferred by seeds; this assembly mechanism is relevant for the coevolution of plants and microorganisms, and critical for potential community responses to environmental changes. We found a specific set of microorganisms present in all *M. senegalensis* plants but not in the surrounding soil, pointing to the presence of a core microbiota in this species. Microbial communities in the shrub understory contributed to enhance plant performance; i.e., by influencing germination rate. The assembly of understory microbial communities is relevant for the coevolution of plants and microorganisms, and critical for potential community responses to environmental changes.

## Data Availability

The datasets generated for this study can be found in the NCBI bioproject PRJNA1223696 (http://www.ncbi.nlm.nih.gov/bioproject/1223696). Code and templates are available at GitHub (https://github.com/Elenadisa/M.senegalensis-core-microbiota).

## Footnotes

1
http://imr.bio/protocols.html

2
http://www.ncbi.nlm.nih.gov/bioproject/1223696
