# Supplementary material for: Soil microbial communities in contrasting environments show a common core of species linked to Maytenus senegalensis shrubs
Source: Front Microbiol. 2026 Jan 27;16:1699694. doi: 10.3389/fmicb.2025.1699694 (PMC12886493; doi:10.3389/fmicb.2025.1699694)

Supplementary Material

Supplementary Table 1. Number of genera identified per experimental condition. The table presents the total number of genera detected in each microhabitat and country, without considering their abundances. Letters indicates if the differences are significant after performing an ANOVA test and a Tukey test.

**PROKARYOTE**				
**Country**	**Microhabitat**	**Genus Number (average)**	**SD**	**Tukey**
Senegal	Gap	96.7	14.9	b
Senegal	Understory	97.4	15.9	b
Spain	Gap	70.1	25.3	a
Spain	Understory	67.1	12.3	a
**FUNGI**				
**Country**	**Microhabitat**	**Genus Number (average)**	**SD**	**Tukey**
Senegal	Gap	151.9	16.5	b
Senegal	Understory	147.0	20.4	b
Spain	Gap	94.4	21.6	a
Spain	Understory	114.1	10.7	a

Supplementary Table 2. PERMANOVA for prokaryote community.

Permutation test for adonis under reduced model
Permutation: free
Number of permutations: 999

adonis2(formula = bray_distance ~ Country * Microhabitat, data = meta, permutations = 999, method = distance)

```
         Df SumOfSqs     R2      F Pr(>F)
Model     3   5.5811 0.4574 9.8347  0.001 ***
Residual 35   6.6208 0.5426
```

Total 38 12.2019 1.0000

Signif. codes: 0 '***' 0.001 '**' 0.01 '*' 0.05 '.' 0.1 ' ' 1

Supplementary Table 3. PERMANOVA for fungi community.

Permutation test for adonis under reduced model
Permutation: free
Number of permutations: 999
adonis2(formula = bray_distance ~ Country * Microhabitat, data = meta, permutations = 999, method = distance)

	Df	SumOfSqs	R2	F	Pr(>F)	
Model	3	6.0142	0.39414	7.5898	0.001	***
Residual	35	9.2448	0.60586			
Total	38	15.2590	1.00000			

Signif. codes: 0 '***' 0.001 '**' 0.01 '*' 0.05 '.' 0.1 ' ' 1

1.1 Supplementary Figures

Supplementary Figure 1. Top 20 most abundant genera (A) Prokaryote. (B) Fungi.

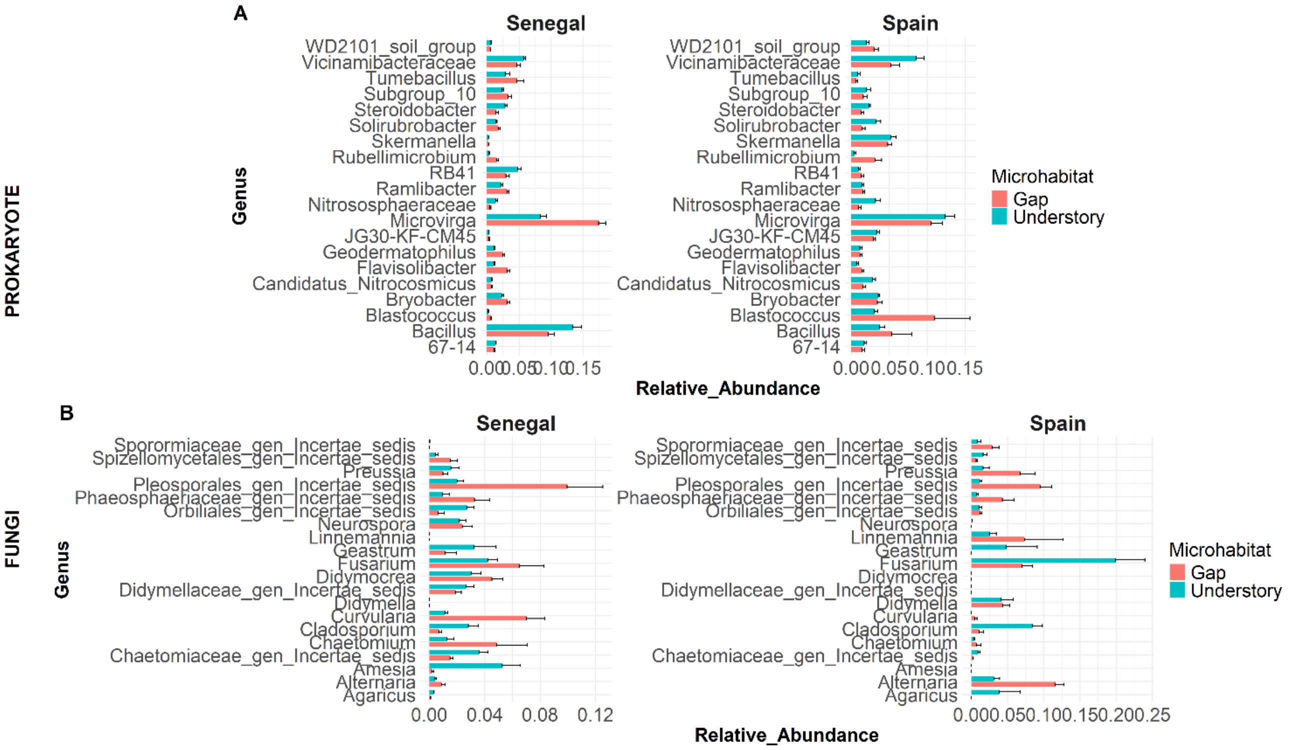

Supplementary Figure 2. Compare relative abundance between understory (x-axis) and gap (y-axis) samples. Points above red line means relative abundance is higher in gap, while points below the red line correspond to genera with a relative abundance higher in understory. Green lines correspond to

understory abundance filter (≥0.2%) and blue line correspond to gap abundance filter (≥0.2%). (A) For prokaryote. (B) For fungi.

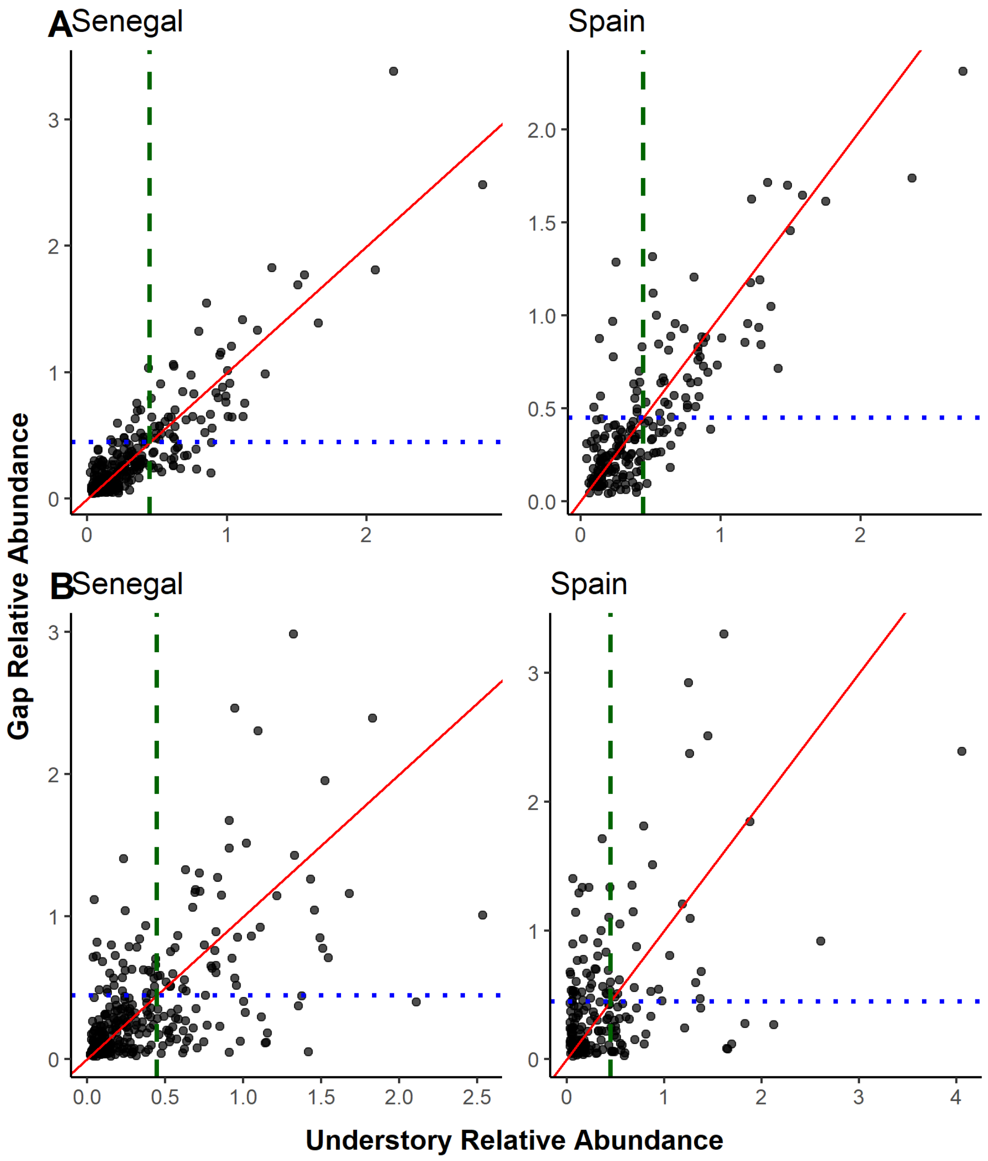

Supplement: Supplementary file 1 [file Data_Sheet_1.PDF]
